# Improvement of Vascular Function by Acute and Chronic Treatment with the GPR30 Agonist G1 in Experimental Diabetes Mellitus

**DOI:** 10.1371/journal.pone.0038787

**Published:** 2012-06-05

**Authors:** Zi-lin Li, Jin-cheng Liu, Shui-bing Liu, Xiao-qiang Li, Ding-hua Yi, Ming-gao Zhao

**Affiliations:** 1 Department of Cardiovascular Surgery, Xijing Hospital, School of Pharmacy, Fourth Military Medical University, Xi'an, China; 2 Department of Pharmacology, School of Pharmacy, Fourth Military Medical University, Xi'an, China; 3 Department of Cardiothoracic Surgery, PLA 452 Hospital, Chengdu, Sichuan, China; University of Padova, Italy

## Abstract

The G-protein coupled estrogen receptor 30 (GPR30) is a seven-transmembrane domain receptor that mediates rapid estrogen responses in a wide variety of cell types. This receptor is highly expressed in the cardiovascular system, and exerts vasodilatory effects. The objective of the present study was to investigate the effects of GPR30 on vascular responsiveness in diabetic ovariectomized (OVX) rats and elucidate the possible mechanism involved. The roles of GPR30 were evaluated in the thoracic aorta and cultured endothelial cells. The GPR30 agonist G1 induced a dose-dependent vasodilation in the thoracic aorta of the diabetic OVX rats, which was partially attenuated by the nitric oxide synthase (NOS) inhibitor, nitro-L-arginine methylester (L-NAME) and the GPR30-selective antagonist G15. Dose-dependent vasoconstrictive responses to phenylephrine were attenuated significantly in the rings of the thoracic aorta following the acute G1 administration in the diabetic OVX rats. This effect of G1 was abolished partially by L-NAME and G15. The acute administration of G1 increased significantly the eNOS activity and the concentration of NO in the endothelial cells exposed to high glucose. G1 treatment induced an enhanced endothelium-dependent relaxation to acetylcholine (Ach) in the diabetic OVX rats. Further examination revealed that G1 induced vasodilation in the diabetic OVX rats by increasing the phosphorylation of eNOS. These findings provide preliminary evidence that GPR30 activation leads to eNOS activation, as well as vasodilation, to a certain degree and has beneficial effects on vascular function in diabetic OVX rats.

## Introduction

A novel estrogen receptor GPR30, which belongs to the family of seven-transmembrane G-protein-coupled receptors, exhibit various rapid biological responses to estrogen [1]. GPR30 has recently been identified to be expressed in the vascular system [2], [3]. Recent studies have demonstrated that the GPR30-specific agonist G1 [4] and the GPR30-selective antagonist G15 [5], which are mainly used to study the role of GPR30 in the cardiovascular system [2], elicits the relaxation of the carotid artery [3], and G1 infusion results in an acute reduction in arterial blood pressure [6]. In addition, vasorelaxation is dependent on endothelium-derived nitric oxide (NO) [3], [7]. GPR30 also plays a critical role in improving glucose-stimulated insulin release while suppressing glucagon and somatostatin secretion [8], [9]. These effects may help reveal a potential novel therapeutic strategy for vascular dysfunction in diabetes mellitus.

Endothelial cells provide a functional barrier and modulate several signals involved in vasomotion. A number of clinical trials for diabetes mellitus have shown that hyperglycemia is a major causal factor in the development of endothelial dysfunction [10]. The slow reactions of hyperglycemia to endothelial cells results in insufficient NO and impairment of endothelium-dependent vasodilation [11].

Endothelial production of NO is critical to the regulation of vascular responses, including regional blood flow and vascular tone, vascular smooth muscle cell proliferation, platelet adhesion, and aggregation and leukocyte-endothelial interactions [12]. eNOS knockout mice show several phenotypes [12], including the lack of endothelium-derived relaxing factor activity and hypertension [13], increased platelet aggregation [14], increased vascular smooth muscle cell proliferation [15], atherosclerosis [16], increased propensity to thrombosis, and leukocyte-endothelial adhesion [17].

Hence, the aim of the present study was to explore whether G1 administered at the same dose as previously described that induced dose-dependent relaxations at aorta [6], carotid [3] and mesenteric arteries [7] can also induce vasodilatory effects on the thoracic aorta with vascular endothelial dysfunction in a diabetic condition and prevent the vascular endothelial dysfunction observed in ovariectomized (OVX) diabetic rats. Moreover, the aim of the present study was to examine the effect of G1 replacement on vascular function in diabetic OVX rats.

## Results

### Effect of GPR30 activation on metabolic parameters

**Figure 1:**
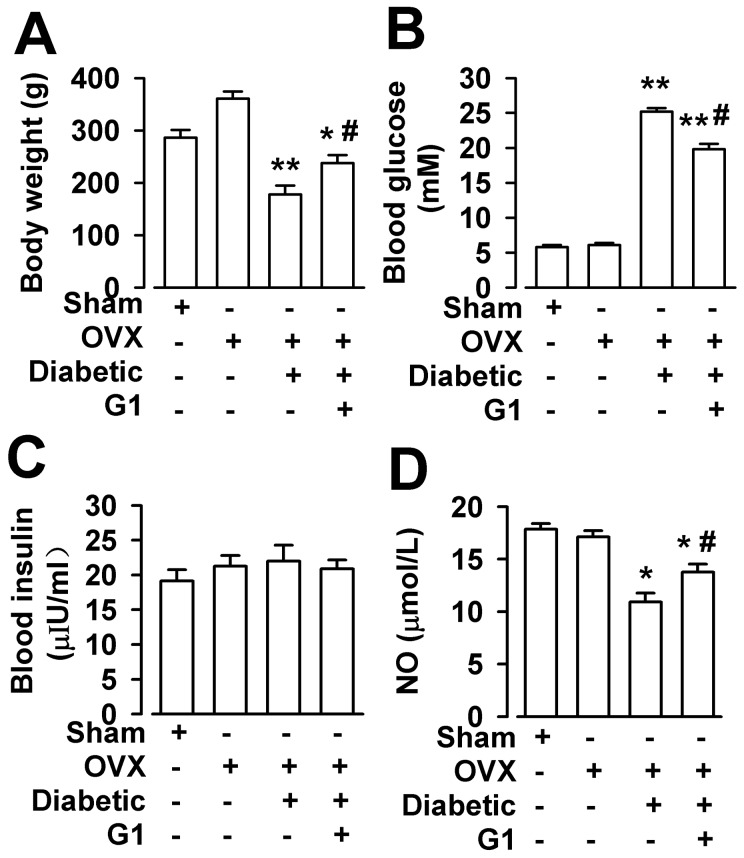
Effects of G1 on body weight, blood glucose, serum insulin and serum NO of diabetic OVX rats. (**A**) Body weight was significantly decreased in OVX rats treated with STZ (n = 6) as compared with those in OVX rats (n = 20). G1 (200 µg/kg, i.p., 2 times per day, 8 weeks) administration (n = 6) attenuated the body weight loss in OVX rats treated with STZ. (**B**) Blood glucose was increased in OVX rats treated with STZ (n = 6) as compared with those in OVX rats (n = 20). G1 administration (n = 6) decreased the high blood glucose impairment in OVX rats treated with STZ. (**C**) The serum insulin levels had no difference among the four groups. (**D**) Diabetic OVX rats (n = 6) display a decrease in NO serum levels compared with those in OVX rats (n = 20). G1 administration (n = 6) increased NO serum levels compared with diabetic OVX rats. ^*^ *p*<0.05, ^**^ *p*<0.01 compared with OVX rats; ^#^ *p*<0.05 compared with diabetic OVX rats.

Although all rats were weight-matched at the beginning of the experiment, the body weights of the diabetic OVX rats were less than those of the normal OVX rats. G1 treatment (200 µg/kg, i.p., 2 times per day, 8 weeks) increased significantly the body weights of the diabetic OVX rats (Fig. 1A). The plasma glucose levels increased significantly in the diabetic OVX rats compared with those in the OVX rats during the 8 weeks period. However, G1 treatment reduced the plasma glucose level in the diabetic OVX rats (Fig. 1B). The serum insulin levels of four groups was not significantly different (Fig. 1C), although, the serum insulin levels was increased slightly in diabetic OVX rats. However, diabetic OVX rats display a decrease in NO serum levels compared with OVX rats. G1 treatment increased NO serum levels compared with the diabetic OVX rats (Fig. 1D).

### Activation of GPR30-induced relaxation in the thoracic artery of the OVX and diabetic OVX rats

**Figure 2:**
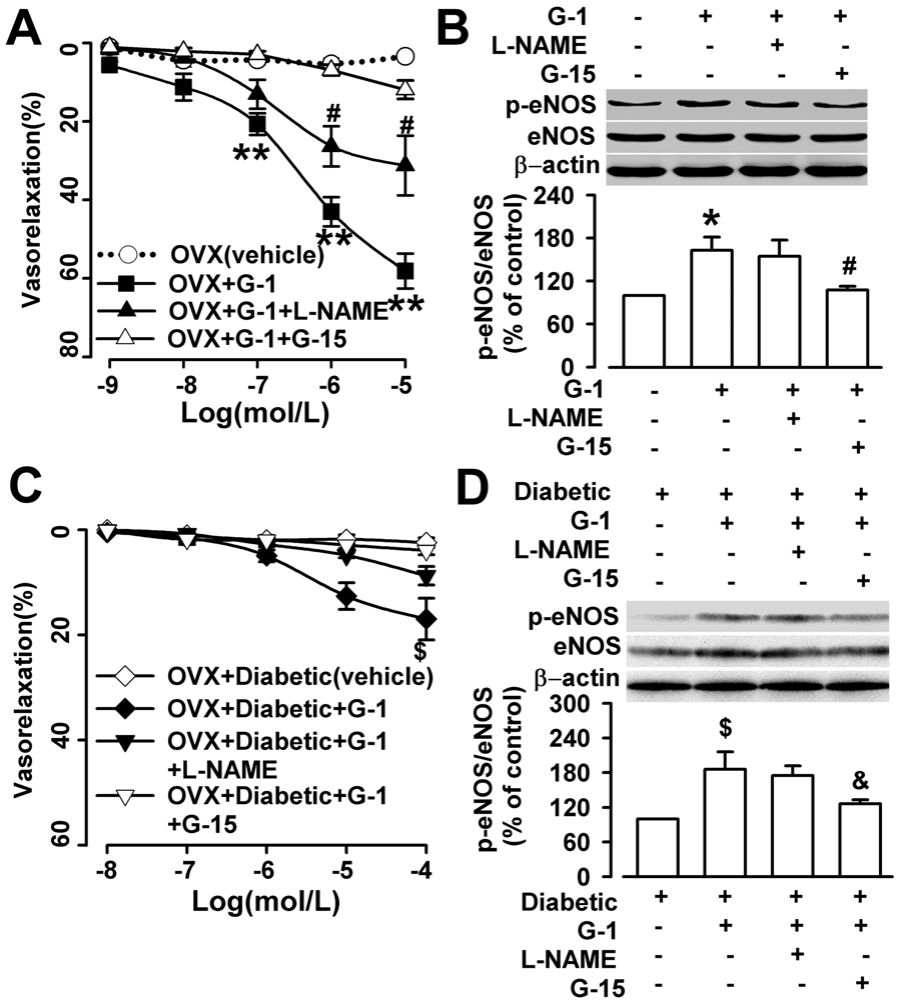
G1-induced relaxation in the thoracic artery in a dose-dependent manner. (**A**) G-1 (1 nM -10 µM) dilated the arteries from the OVX rats in a dose-dependent manner; however, L-NAME (100 µM) and G15 (1 µM) partially abolished the effect of G1. n = 6 rings from 3 rats. (**B**) G1 increased the phosphorylation level of eNOS (p-eNOS; at serine1177) in arteries from OVX rats, and the phosphorylation level of eNOS induced by G1 was partially attenuated by G15. (**C**) G-1 (10 nM -100 µM) dilated the arteries from the diabetic OVX rats in a dose-dependent manner; however, L-NAME (100 µM) and G15 (1 µM) abolished the effect of G1. n = 6 rings from 3 rats. (**B**) G1 increased the phosphorylation level of eNOS (p-eNOS; at serine1177) in arteries from diabetic OVX rats, and the phosphorylation level of eNOS induced by G1 was partially attenuated by G15. ^*^ *p*<0.05 compared with OVX rats; ^#^ *p*<0.05 compared with OVX+G1 rats; ^$^ *p*<0.05 compared with diabetic OVX rats; ^**^ *p*<0.01 compared with OVX rats; ^&^ *p*<0.05 compared with diabetic OVX+G1 rats. Results are given as the mean ± SEM of three independent experiments.

Bath application of G1 (1 nM -10 µM) induced strong relaxation of the aortic rings from the OVX rats in a dose-dependent manner (Fig. 2A). And, G1 also induced relaxation of the aortic rings from the diabetic OVX rats in a dose-dependent manner (Fig. 2C). The effect of G1 in the absence or presence of the NOS inhibitor L-NAME (100 µM) and the GPR30-selective antagonist G15 (1 µM) [5], [18] were detected to identify the role of NO on GPR30-mediated vasorelaxation. The vascular relaxation induced by G1 was partly abolished by L–NAME and abolished by G15 in the aortic strips from the OVX and diabetic OVX rats (Fig. 2A, C). Last, western blot analysis was performed in these aortic rings. Bath application of G1 increased significantly the phosphorylation level of eNOS in the aortic strips from the OVX and diabetic OVX rats, and the phosphorylation level of eNOS induced by G1 was also attenuated by G15 (Fig. 2B, D).

### Effect of GPR30 activation on the contraction response to phenylephrine (PE) in the thoracic arteries of the diabetic OVX rats

**Figure 3:**
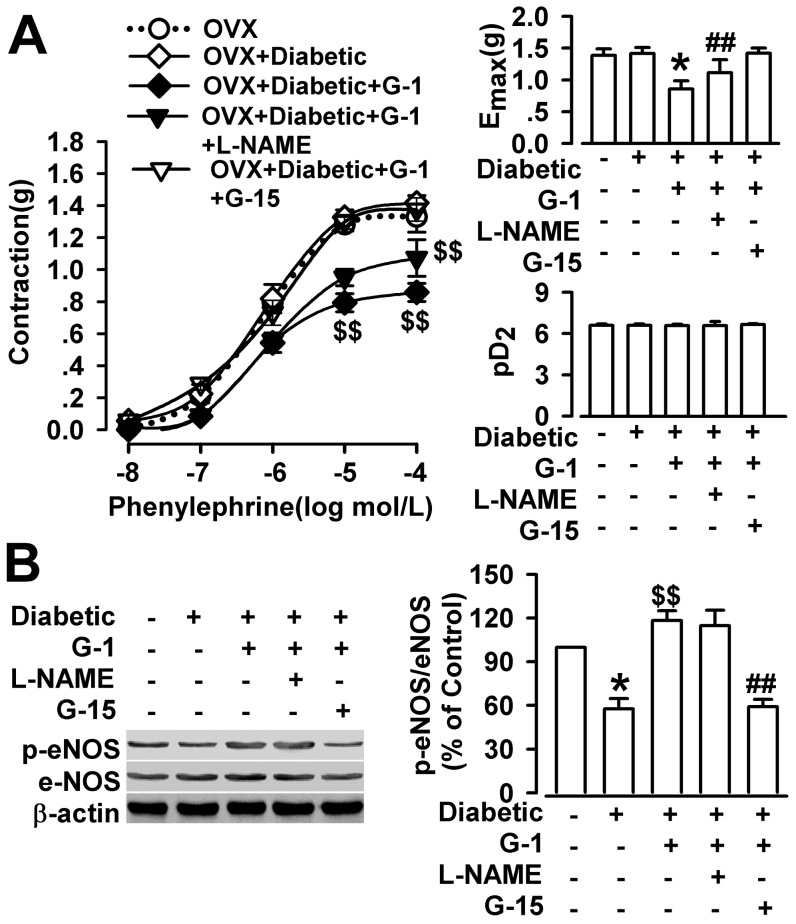
G1 pretreatment on the contraction response to PE in diabetic OVX rats. (**A**) There was no difference on contraction of thoracic aorta between OVX and diabetic OVX groups. G1 (3 µmol/L) treatment dilated the thoracic arteries in the presence of PE (1 mM) in diabetic OVX rats. L–NAME (100 µM) and G15 (1 µM) abolished the effect of G1 in the diabetic OVX rats as shown by the maximal response (E_max_) to PE. The sensitivity of arteries to PE (pD_2_) did not change among groups. (**B**) The eNOS phosphorylation was significantly reduced in OVX diabetic group as compared with OVX group. G1 treatment increased the eNOS phosphorylation; whereas, G15 blocked the eNOS phosphorylation induced by G1. n = 6–12 rings from 4–6 rats.^*^ *p*<0.05 compared with OVX rats; ^##^ *p*<0.01 compared with diabetic OVX+G1 rats; ^$$^ *p*<0.01 compared with diabetic OVX rats. Results are given as the mean ± SEM of three independent experiments.

In present study, the maximum constriction of PE of the aortic rings from the OVX rats is slightly lower than that of diabetic OVX rats. But, no significant difference was found in either the sensitivity to or the maximum constriction of PE (1 mM) between the OVX and diabetic OVX rats, which indicated that diabetes had no significant effect on the vasoconstrictor responses of aortic segments to PE in our experiment (Fig. 3A). G1 (3 µmol/L) treatment dilated significantly the thoracic arteries in the presence of PE, compared with the OVX or diabetic OVX group (Fig. 3A).

To explore the possible mechanism underlying the G1-induced vasoactive response, we examined the transient vasoconstrictor response to PE in the presence of L–NAME (100 µM) and G15 (1 µM). The vascular relaxation induced by G1 in the presence of PE was partly abolished by L–NAME and abolished by G15 in the diabetic OVX rats (Fig. 3A). Western blot showed that diabetes depressed markedly the phosphorylation level of eNOS in the OVX rats. However, G1 treatment raised significantly the phosphorylation level of eNOS, compared with the OVX or diabetic OVX rats, and G15 blocked the effect of G1 on eNOS phosphorylation (Fig. 3B).

### Effect of chronic G1 administration on the relaxation response to ACh or SNP in the thoracic arteries of the diabetic OVX rats

**Figure 4:**
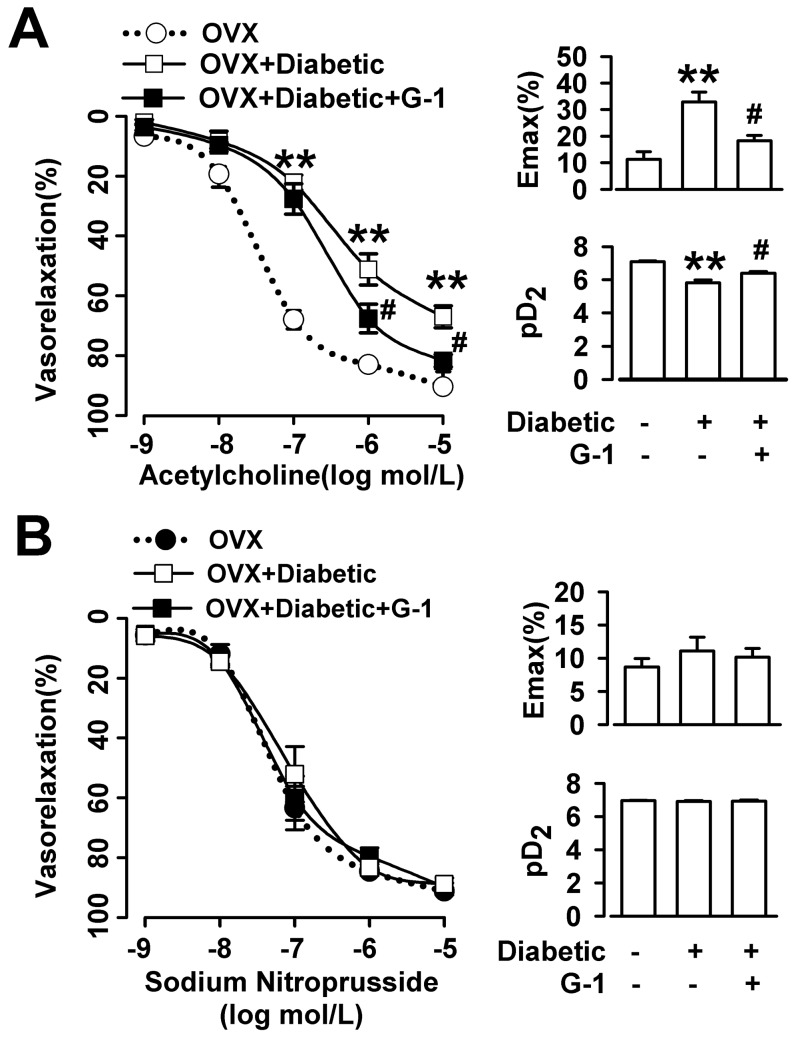
Effect of G1 on the relaxation response to ACh or SNP. (**A**) Capability of the relaxation induced by ACh (10^−9^ M to 10^−5^ M) was reduced in diabetic OVX rats as compared with OVX rats. Long-term administration of G1 (200 ug/kg i.p., 2/per day, 8 weeks) increased the capability of vasorelaxation as shown by the E_max_ and pD_2_. (**B**) The capability of the relaxation caused by SNP (10^−9^ M to 10^−5^ M) had no difference in the aortic segments among the three groups. n = 6–12 rings from 5–8 rats. ^**^ *p*<0.01 compared with OVX rats; ^#^ *p*<0.05 compared with diabetic OVX rats. Results are given as the mean ± SEM of three independent experiments.

When the PE-induced contraction had reached a plateau in organ chambers, ACh (10^−9^ M to 10^−5^ M) or SNP (10^−9^ M to 10^−5^ M) was added cumulatively. The capability of the concentration-dependent relaxation induced by ACh, with the maximum response at 10^−5^ M, was weaker in the aortic segments from the diabetic OVX rats than those from the OVX rats (Fig. 4A). After the chronic administration of G1 (200 ug/kg p.i. 2/per day), the capability of ACh-induced relaxation in the aortic segments from the diabetic OVX rats was enhanced significantly. The capability of the relaxation caused by SNP (10^−9^ M to 10^−5^ M) had no difference in the aortic segments among the three groups (Fig. 4B).

### The expressions of GPR30 and eNOS protein in endothelial cells and aortas

**Figure 5:**
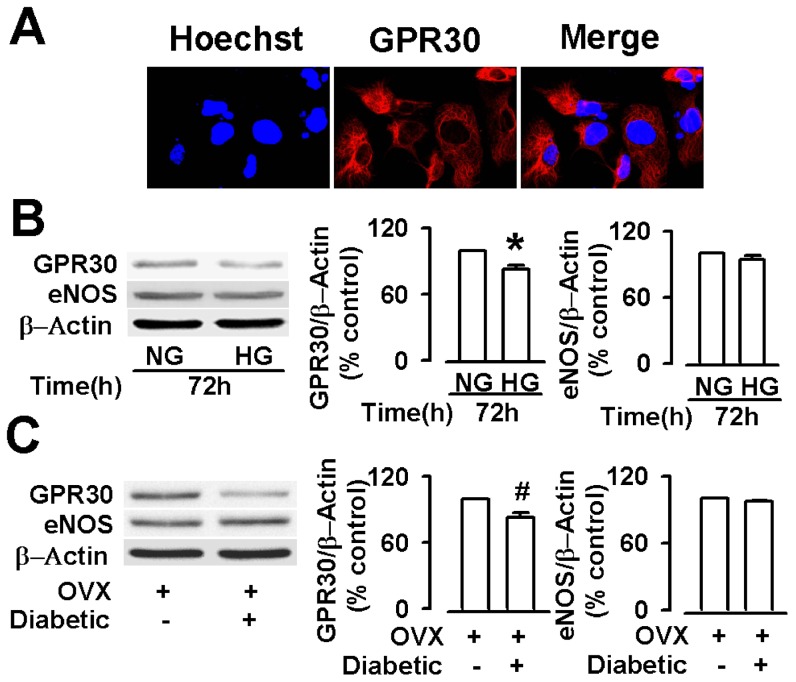
The expressions of GPR30 and eNOS protein in endothelial cells and aortas. (**A**) GPR30 expression in endothelial cells from intact SD female rats (red = GPR30). (B) Western Blot analysis of GPR30 and eNOS expression showed that cultures of the endothelial cells maintained in high glucose (HG, 33 mM) showed reduction in GPR30 expression compared to normal glucose (NG, 5 mM). (C) Western Blot analysis of GPR30 and eNOS expression also showed reduction in GPR30 expression in the aortas from diabetic OVX rats compared to those from diabetic OVX rats. ^*^ *p*<0.05 compared with NG; ^#^ *p*<0.05 compared with OVX rats. Results are given as the mean ± SEM of three independent experiments.

The endothelial cells displayed a positive immunostaining for GPR30 (Fig. 5A). We next examined the expressions of GPR30 and eNOS in the endothelial cells by Western blotting. In endothelial cells, the GPR30/β-actin ratio was decreased in the high glucose media for 72 h, compared with that in the samples in the low glucose media (Fig. 5B). In addition, we also evaluated the expression of GPR30 and eNOS protein in aortas from OVX and diabetic OVX rats. The expression of the GPR30 was also decreased in aortas from diabetic OVX rats (versus OVX rats) (Fig. 5C). The expression of the eNOS protein was not changed in endothelial cells and aortas (Fig. 5B, C).

### Effect of G1 on eNOS enzymatic activity in endothelial cells

**Figure 6:**
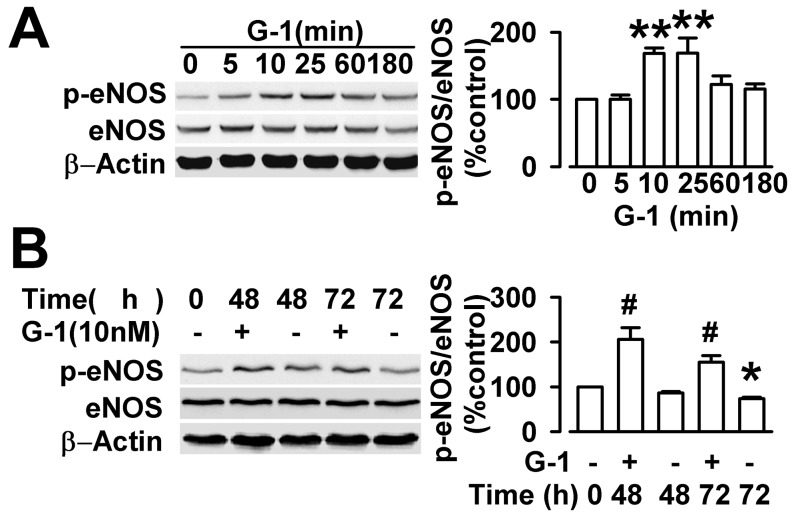
Effect of G1 on eNOS enzymatic activity in endothelial cells. (**A**) The endothelial cells were cultured in vitro, and treated with G1 (10 nM) for 5, 10, 25, 60, 180 min. G1 activated eNOS phosphorylation after 5–10 min treatment. The increased level was maintained up to 30 min, followed by a reduction at 1 h. ^**^ *p*<0.01 compared with control. (**B**) Endothelial cells were exposed to high glucose concentration (33 mM) for 48 or 72 h, and then incubated with G1(10 nM) for 10 min. G1 treatment (10 nM, 10 min) reversed the down-regulation of phosphorylation level of eNOS by high concentration of glucose. ^*^ *p*<0.05 compared with the glucose treated before; ^#^ *p*<0.05 compared with the glucose treated at 48 h or 72 h without G1. Results are given as the mean ± SEM of three independent experiments.

**Figure 7:**
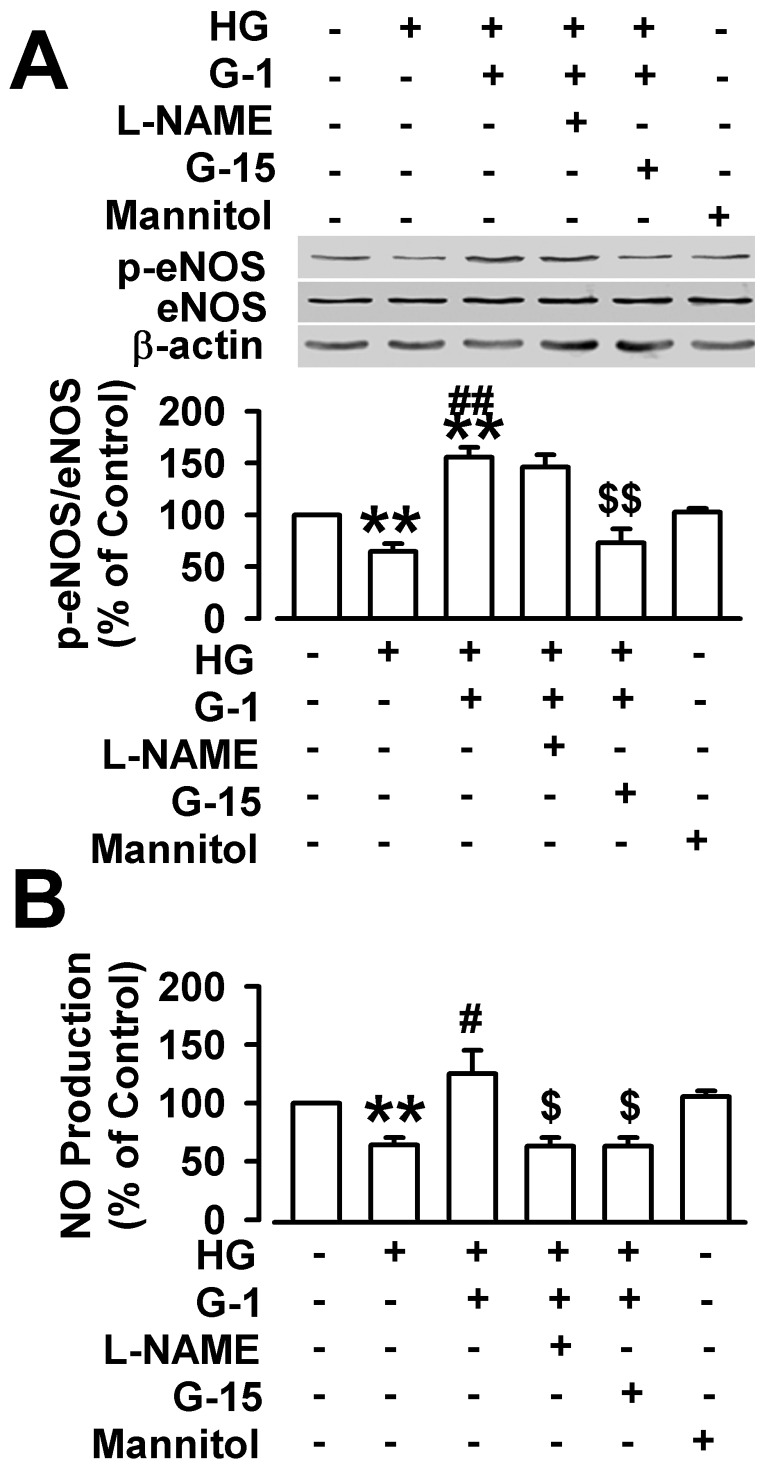
Activation of eNOS signaling pathway mediated protective effects of G1. The endothelial cells were treated with high glucose (HG) for 72 h, and then treated with L-NAME (100 µM) for 40 min and G15 (1 µM) or without them in presence of G1 (10 nM) for 10 min. (**A**) G1 treatment (10 nM, 10 min) reversed the down-regulation of phosphorylation level of eNOS by high concentration of glucose. This effect was blocked by G15. (**B**) G1 reversed the NO reduction in endothelial cells exposure to HG. This effect of G1 was blocked by L-NAME and G15. High concentration of mannitol (33 mM) did not alter the phosphorylation level of eNOS. ^**^ *p*<0.01 compared with the glucose treated before; ^#^ *p*<0.05, ^##^ *p*<0.01 compared with the HG treated; ^$^ *p*<0.05, ^$$^ *p*<0.01 compared with the HG+G1 treated. Results are given as the mean ± SEM of three independent experiments.

The effect of G1 on eNOS activation was examined to explore the potential signaling pathways contributory to the G1-evoked vasodilation. The cultured endothelial cells treated with G1 (10 nM) in serum-free medium. The amounts of phosphorylated eNOS in endothelial cells were detected by Western blot (Fig. 6A). G1 activated the eNOS phosphorylation after a 5 min to 10 min treatment. The increased level was maintained up to about 30 min, followed by a reduction at 1 h (Fig. 6A). We also investigated the influence of G1 on the activation of eNOS in the cultured endothelial cells exposed to high glucose. The phosphorylation levels of eNOS decreased significantly when the endothelial cells were treated with 33 mM of glucose for 48 h or 72 h (Fig. 6B). However, high concentration of mannitol (33 mM) did not alter the phosphorylation level of eNOS (Fig. 7A). Following the high glucose treatment, application of G1 (10 nM, 10 min) reversed the down-regulation of phosphorylation level of eNOS by high concentration of glucose (Fig. 6B; Fig. 7A).

### Effect of NOS inhibitor and GPR30-selective antagonist on the NO release in endothelial cells

The possible involvement of GPR30 in the NO release in the endothelial cell was determined by preincubating the endothelial cells in high glucose for 48 h, followed by treatment with L–NAME (100 µM) for 40 min or without L-NAME, or with G15 for 15 min or without G15, and thereafter treated with G1 (10 nM) for 10 min or without G1. The phosphorylation level of eNOS decreased significantly in the endothelial cells exposed to high glucose. G1 increased evidently the phosphorylation level of eNOS of endothelial cells in the presence of high glucose. The effect of G1 on eNOS phosphorylation was blocked by G15 (Fig. 7A). In addition, the NO release was detected by Nitric Oxide Assay Kit (Fig. 7B). NO production decreased significantly in the endothelial cells exposed to high glucose. G1 evidently increased the NO production of the endothelial cells in the presence of high glucose. The effect of G1 on NO production was blocked by L-NAME and G15 (Fig. 7B). High concentration of mannitol (33 mM) did not alter the NO production (Fig. 7B).

## Discussion

The present study yielded the following novel findings: (1) G1 treatment reduced blood glucose and ameliorated the diabetes-induced loss in body weight and NO serum levels in the diabetic OVX rats; (2) Administration of G1 for 8 wk could partially prevent the functional changes on vascular reactivity in the diabetic OVX rats; (3) The dose-dependent vasoconstrictor responses to PE are significantly attenuated following the acute G1 administration in the diabetic OVX rats; and (4) The activation of GPR30 induced the phosphorylation of eNOS, which enhanced the NO-dependent vasodilation. The results suggest that the activation of GPR30 activation has the potential to improve the vascular function in diabetes.

The body weights of the diabetic OVX rats were less than those of the OVX rats. G1 treatment increased the body weights, decreased blood glucose and ameliorated nitric oxide serum levels in type 2 diabetic rats induced by a high-fat diet combined with low doses of streptozotocin. These findings suggest that G1 has an antidiabetic effect. Recent studies have shown that E2 and G-1 improved insulin secretion [8], [9], [19]. The mechanism of the antidiabetic action may be through activation of the epidermal growth factor receptor and ERK [8], and anti-apoptotic effects in pancreatic islets [19] by GPR30. In addition, depression of glucagon and somatostatin secretion may also play a critical role in the secretion of insulin [9].

Endothelial production of NO is critical to the regulation of vascular responses. The reduction in the activity of eNOS may result in the lower production of NO. It is also known that the phosphorylation of eNOS contributes to the regulation of eNOS activity [20]. Among the regulatory eNOS phosphorylation sites, Ser^1177^ is the one that is most extensively studied [20]. And, a lot of stimuli that promote eNOS activation can cause phosphorylation of this site. Both basal and stimulated NO synthesis will be reduced by preventing eNOS phosphorylation at Ser^1177^ by mutating the site to alanine [21]. Diabetes mellitus is associated with the endothelial dysfunction and bioavailabilty of NO. The phosphorylation level of eNOS was detected to explore further the molecular mechanisms of GPR30. eNOS activity depends on eNOS phosphorylation, which Ser^1,177^ has characterized extensively [22]. G1 activated eNOS phosphorylation and NO production in the endothelial cells. L-NAME or G15 not only attenuated the effect of G1 on the vasodilation of the thoracic aorta, but also reduced the release of NO. In addition, G15 also can reduce G1-evoked eNOS phosphorylation. These findings suggest that the endothelium-dependent relaxation induced by G1 may be caused by the eNOS activity, which is consistent with the study reported by Meyer [23]. In addition, acute G1 administration also dilated the thoracic arteries exposed to PE in the diabetic OVX rats. When the aortic rings of the diabetic OVX rats were incubated with L-NAME and G1, L-NAME abolished partially the effect of G1 in the presence of PE. These results suggest that the enhancement of PE-induced contractile responses in the aortas of the diabetic OVX rats may be caused by a diabetes-related impairment of the endothelial NO generation, and G1 can attenuate this impairment in the endothelial cells through the eNOS signaling pathway. This phenomenon is consistent with the results in several recent studies [2], [3], [6], [7], [24], [25] that have reported that the activation of eNOS phosphorylation is involved in the relaxation of the aortic rings induced by G1.

The results of the current study indicate that G1 induces a vasorelaxation in the thoracic aortas of the diabetic OVX rats, which is blocked partially by the NOS inhibitor(L-NAME) or the GPR30-selective antagonist (G15). Hence, we considered the possibility of partial reversal of endothelial dysfunction by GPR30. The assessment of physiologic vasodilator responses can measure endothelial dysfunction [26]. Previous studies have reported that the forearm blood flow response to ACh is reduced in patients with type 2 diabetes, suggesting endothelial dysfunction [27]–[29]. The results of the present study indicate that ACh-induced vasodilation is markedly blunted in the diabetic OVX rats, but recovered partially after the chronic administration of G1. A preliminary report have indicated that G1 reduces levels of superoxide [3]. Vascular dilation caused by ACh is also reported to depend on the NO produced by the endothelium [12].

In the current study, we report for the first time that G1 increased eNOS phosphorylation in the endothelial cells. Pretreatment with high glucose resulted in a significant decrease in NO, compared with the control. Incubation with G1 could restore NO production. Furthermore, G15 suppressed effectively the G1-induced activation of eNOS and decreased NO production. These results indicate that G1 increases the NO availability via eNOS phosphorylation. Furthermore, the results of the present study confirmed that at least part of the vasodilation of G1 is linked to the release of NO [3], [7].

Insufficient endothelial NO availability is known to be an important mechanism underlying endothelial dysfunction [30], with conditions conducive to the development of atherosclerosis, vasoconstriction, thrombosis, inflammation, and neointimal proliferation [12]. Thus, the reduction in eNOS phosphorylation is an important cause of endothelial dysfunction [31], [32]. Moreover, G1, a selective agonist of GPR30, may improve vascular function by enhancing NO availability. These biological actions support the idea of GPR30 as a vascular protection factor. However, the role of GPR30 in vascular health and its functions or molecular mechanisms need further investigation.

In summary, the activation of GPR30 elicits an endothelium-dependent, NO-mediated vascular relaxation in the thoracic aorta of diabetic OVX rats, and chronic G1 treatment could prevent vascular endothelial dysfunction in the diabetic OVX rats. Given that endothelial NO has a crucial role not only in modulating vascular tone but also in antiatherosclerosis [12], we can speculate that increased NO bioavailability induced by G1 could also participate in the inhibited atherosclerotic process in diabetic OVX rats.

## Materials and Methods

### Animal

Six-week-old Sprague-Dawley female rats weighing approximately 200 g were maintained on a 12 h light/dark cycle at a constant room temperature (22°C±1°C). Bilateral ovariectomy was performed on all animals with 3% pentobarbital sodium anesthesia. To induce diabetes mellitus, the rats were fed with high-fat diet (HFD) after ovariectomy for 2 months, and then were injected intraperitoneally with streptozotocin (STZ, 30 mg/kg) (Sigma-Aldrich, St. Louis, MO, USA), which was dissolved in sodium citrate buffer (pH 4.2). The control rats were fed with normal rodent diet, which were injected with a similar volume of citrate buffer. The composition of the normal rodent diet was 20% protein and 4.5% fat. The HFD diet was composed of 21.2% protein, 12% fat, 15% sucrose, and 1% cholesterol. The blood glucose concentrations were measured by a glucometer (Accutrend; Bayer, Mannheim, Germany) 48 h post-STZ injection, and rats with blood glucose ≥13 mM were considered diabetic. Insulin was measured by radioimmunoassay kit (BNIBT, Beijing, China). Serum NO levels were assayed by NO detection kit (Nanjing Jiancheng Bioengineering Institute, China). The current study was performed in adherence to the National Institutes of Health guidelines for the use of experimental animals, and all animal protocols were approved by the Committee for Ethical Use of Experimental Animals of the Fourth Military Medical University.

### Vascular reactivity

The animals were anesthetized by the intraperitoneal administration of 20% urethane 8 wk post-injection of STZ or buffer. Aorta from the heart to the iliac bifurcation was removed and placed in ice-cold Krebs buffer consisting of (mM): NaCl, 118; KCl, 4.8; CaCl_2_·2H_2_O, 2.5; MgCl_2_·6H_2_O, 2.5; NaH_2_PO_4_·2H_2_O, 1.2; NaHCO_3_, 8.5; and glucose·H_2_O, 11. The aorta was cleared of fat, as well as connective tissue, and cut into rings 2 mm long. The rings were mounted onto hooks, suspended in organ chambers filled with Krebs buffer, aerated with 95% O_2_/5% CO_2_ at 37°C, and connected to pressure transducers (WPI, Sarasota, FL) to record the changes via a Mac-Lab recording system. After 30 min of equilibration at an optimal preload of 9.8 mN, the rings were stimulated with 1 mM of phenylephrine (PE, Sigma-Aldrich, St. Louis, MO, USA) and 1 mM of acetylcholine (Ach, Sigma-Aldrich, St. Louis, MO, USA) (both from Sigma-Aldrich, St. Louis, MO), and rings with >50% relaxation were considered endothelium-intact. Nitro-L-arginine methylester (L-NAME, 100 µM, Beyotime Institute of Biotechnology, China) [7] and the GPR30-selective antagonist G15 (1 µM, Calbiochem, Germany) [7] were used to pretreat the vessels of the OVX rats to confirm the role of GPR30 on the vascular function of rats [3], [7]. Since G15 was prepared in dimethylsulfoxide (DMSO), DMSO was used in all groups with the same volume in G15 group. The rings were treated as follows: (1) The roles of eNOS in the GPR30-mediated relaxation were assessed by plotting concentration-response curves of G1 (1 nM to 100 µM, Cayman Chemical, Ann Arbor, MI) or the vehicle dimethylsulfoxide (DMSO) in the rings pretreated with or without L-NAME and G15 in organ chambers; (2) Prior to the detection of the PE dose-response curves in the rings of OVX and diabetic OVX rats, some of rings from diabetic OVX rats were first incubated with L-NAME (100 µM) or G15 (1 µM) for 20 min and then with G1 (3 µM) for 10 min in organ chambers; (3) The roles of GPR30 in the vascular function were assessed in the diabetic OVX rats treated with G1 (200 µg/kg, i.p., 2 times per day) for 8 weeks. The aortic rings were preconstricted with PE in organ chambers, and the vasorelaxation responses were evaluated with increasing doses of ACh (10^−9^ mol/L to 10^−5^ mol/L). The maximal effective tension (E_max_) was determined with the vascular response to PE. The maximal vasomotor response was expressed as a percentage of the contraction induced by 1 mM of PE (%R_max_ for relaxation), and the vascular sensitivity was expressed as pD_2_ (−logEC50).

### Cell culture and treatments

The aortic endothelial cells (Product ID: RAT-CELL-0008; PriCell) were maintained in RPMI 1640 medium (HyClone, UT, USA) supplemented with heat-inactivated fetal bovine serum (10%), 2 mM of L-glutamine, 100 U/mL of penicillin, and 100 g/mL of streptomycin. Then, the cells were incubated at 37°C in 5% CO_2_ and 95% air. G1 stock solution was prepared in DMSO and diluted with culture medium immediately prior to the experiment, and 0.01% DMSO was used as a sham control. The cells were incubated in serum-free medium for 24 h, and treated with different concentrations of G1 (10 nM) [33], L-NAME (100 µM) [34], G15 (1 µM) [35], high glucose (33 mM) and mannitol (33 mM) at different intervals in the same medium, after which they were harvested for further analysis. NO levels in culture medium was assayed by NO detection kit (Nanjing Jiancheng Bioengineering Institute, China).

### Western blot analysis

As described previously [36], equal amounts of proteins (30 µg) from the cultures or the aortas were separated and electrotransferred onto the NC membranes (Invitrogen, Carlsbad, USA) that were probed with anti-eNOS (dilution ratio 1∶1000; Cell signaling, Danvers, MA), anti-phospho-eNOS (Ser^1177^, dilution ratio 1∶1000; Cell signaling), Anti-GPR30 antibody (1∶250; Abcam) and β-actin (dilution ratio 1∶10000; Sigma-Aldrich, St. Louis, MO) antibodies diluted in blocking buffer. Each primary antibody was incubated overnight at 4°C. After 3 washes with Tris-Buffered Saline and Tween 20 for approximately 15 min, the membranes were incubated with horseradish peroxidase-conjugated secondary antibodies (anti-rabbit/anti-mouse immunoglobulin G for the primary antibodies), and the bands were visualized using an ECL system (PerkinElmer). The data were pooled from three independent experiments.

### Immunocytochemistry

The cultured endothelial cells were fixed with ice-cold 4% paraformaldehyde in PBS (pH 7.4) for 30 min, blocked with 5% BSA in PBS for 2 h, incubated overnight with primary anti-GPR30 (1∶300) at 4°C, and then incubated with Cy3-conjugated goat anti-rabbit IgG (Sigma) diluted to 1∶100 in blocking solution. Coverslips were mounted onto slides with 50% glycerin. The stained samples were photographed and were analyzed using an Olympus Fluoview FV100 (Olympus, Japan).

### Statistical analysis

Data are expressed as mean ± SEM. Statistical comparisons were performed using *t*-test, and the differences between the multiple groups were assessed using one-way ANOVA. *p*<0.05 was considered statistically significant.
